# Activation timing of postural muscles of lower legs and prediction of postural disturbance during bilateral arm flexion in older adults

**DOI:** 10.1186/s40101-017-0160-8

**Published:** 2017-12-22

**Authors:** Chie Yaguchi, Katsuo Fujiwara, Naoe Kiyota

**Affiliations:** 1 Department of Rehabilitation, Japan Health Care College, 6-17-3 Megumino-nishi, Eniwa, 061-1373 Japan; 2 Department of Sports and Health, Kanazawa Gakuin University, 10 Sue-machi, Kanazawa, 920-1392 Japan; 3 Department of Rehabilitation, Japan Health Care College, 6-17-3 Megumino-nishi, Eniwa, 061-1373 Japan

**Keywords:** Older adults, Probability, Activation timing of postural muscle, Event-related brain potential, P300, Predictability, Ankle joint

## Abstract

**Background:**

Activation timings of postural muscles of lower legs and prediction of postural disturbance were investigated in young and older adults during bilateral arm flexion in a self-timing task and an oddball task with different probabilities of target presentation. Arm flexion was started from a standing posture with hands suspended 10 cm below the horizontal level in front of the body, in which postural control focused on the ankles is important.

**Methods:**

Fourteen young and 14 older adults raised the arms in response to the target sound signal. Three task conditions were used: 15 and 45% probabilities of the target in the oddball task and self-timing. Analysis items were activation timing of postural muscles (erector spinae, biceps femoris, and gastrocnemius) with respect to the anterior deltoid (AD), and latency and amplitude of the P300 component of event-related brain potential.

**Results:**

For young adults, all postural muscles were activated significantly earlier than AD under each condition, and time of preceding gastrocnemius activation was significantly longer in the order of the self-timing, 45 and 15% conditions. P300 latency was significantly shorter, and P300 amplitude was significantly smaller under the 45% condition than under the 15% condition. For older adults, although all postural muscles, including gastrocnemius, were activated significantly earlier than AD in the self-timing condition, only activation timing of gastrocnemius was not significantly earlier than that of AD in oddball tasks, regardless of target probability. No significant differences were found between 15 and 45% conditions in onset times of all postural muscles, and latency and amplitude of P300.

**Conclusion:**

These results suggest that during arm movement, young adults can achieve sufficient postural preparation in proportion to the probability of target presentation in the oddball task. Older adults can achieve postural control using ankle joints in the self-timing task. However, in the oddball task, older adults experience difficulty predicting the timing of target presentation, which could be related to deteriorated cognitive function, resulting in reduced use of the ankle joints for postural control.

## Background

Deterioration of equilibrium function is considered as a primary cause of falls among older individuals [1]. In several postural tasks needing dynamic equilibrium function, changes in postural control strategy with aging have been reported, particularly in the form of reduced involvement of the ankle joints. For instance, when postural disturbance is induced by transient floor translation [1–4] or a load impact against the subject [5], young adults mainly use the ankle joints for postural control [2, 3, 5], whereas older adults tend to mainly use the hip joints [1, 4, 5]. When older adults voluntarily incline the whole body forward or backward, the lower legs are not inclined in the same direction [1, 6]. In addition to these situations, dynamic postural control is needed during arm movements in daily life.

When arm movements are rapidly executed while standing, the postural muscles of the legs and trunk are automatically activated before the focal muscles of the arms, to moderate any disturbances caused by the arm movements [7]. In older adults, preceding activation of postural muscles is found for the trunk muscles, but is less observable for the thigh muscles, as compared with young adults [8–10]. Furthermore, postural movement during arm movement is smaller at the ankles than at the hips [11, 12]. These previous studies were performed with arm flexion originating from the side of the body to the horizontal level. Preceding activation of the triceps surae has not been observed during such arm flexion [13] even if young adults start the movement at their own pace (self-timing task) [14]. Cordo and Nashner [15] found that preceding muscle activation was clearly observable in the postural muscles playing the most important roles in balance maintenance. These findings suggest that the focus of postural control would not be on the ankle joints during such arm flexion, and few investigations of postural control patterns in older adults have thus focused on the ankle joints during arm movement.

When arm flexion was performed from a position with the hands suspended in front of the body, preceding activation of the triceps surae has been clearly observed [16–18]. In this posture, immediately after the start of arm flexion, the forward moment caused by the weight of the arms acting on the body should be larger in comparison to the posture with arms at the side of the body. Backward inclination of the whole body pivoting at the ankles is suggested to be a postural movement pattern effectively translating the center of gravity backward [19, 20]. Thus, in arm flexion from a suspended position, postural control focused on the ankle joints should be needed to resist rapid forward postural disturbance. When young adults performed this arm flexion, activation timing was unaffected by task condition for the erector spinae (ES), but was earlier in a self-timing task than in reaction tasks for leg postural muscles, especially the triceps surae [16]. In the self-timing task, postural disturbance timing was easily predicted, allowing selection of the most suitable postural control pattern. The postural control pattern during arm movement in older adults was thus first investigated using arm flexion from a suspended position in the self-timing task. Older adults were hypothesized to show a postural control pattern focused not on the ankle joints but rather mainly on the hip joints even in situations where postural control focused on the ankles is important.

In an oddball task, where cognitive function is strongly concerned with task execution, a target or non-target stimulus appears repeatedly in random order at a fixed inter-stimulus interval and the participant is required to perform a specific response to the target stimulus [21–23]. In this task, positivity at approximately 300 ms after target stimulus (P300 component) appears on averaged electroencephalogram (EEG) waveforms recorded from the parietocentral portion [21–23]. P300 is an event-related brain potential and reflects cognitive processing, such as evaluation and judgment of sensory stimuli [24] and subsequent context updating [23, 25]. Latency and amplitude of P300 indicate the cognitive processing time and allocation of attention to the processing, respectively [21–23]. With a higher probability of target presentation, prediction of target appearance is reportedly easier [26], and the latency and amplitude of P300 become shorter and smaller [22, 23, 26, 27]. Easier prediction of target presentation timing with a higher probability could thus result in decreased processing time and decreased allocation of attention to the cognitive processing. When arm flexion from a suspended posture is used as a response action in the oddball task, the relationship between changes in cognitive processing and onset timing of postural muscle activation by predictability could be directly investigated. For young adults, with higher probability, the preceding time of the triceps surae activation would be longer, relating to shorter latency and smaller amplitude of P300.

In contrast, changes in P300 amplitude with increasing target probability (from about 20% to about 80%) were reportedly smaller for older adults than for young adults [26, 28]. This would indicate difficulty in predicting target appearance based on the sequence of target and non-target stimuli and in changing attentional allocation to cognitive processing in proportion to probability. For older adults, the effects of target probability are hypothesized to be less apparent in onset times of postural muscle activation and the latency and amplitude of P300, and onset of the triceps surae activation in oddball tasks would be markedly later than in a self-timing task.

The purpose of this study was to investigate the postural control pattern in older adults focused on the ankle joints during arm movement. For that, older adults performed bilateral arm flexion from a suspended arm position in the self-timing task. Furthermore, to investigate the relationship between anticipatory postural control and cognitive function, this arm movement was carried out in oddball tasks with different probabilities of target presentation.

Our working hypotheses were as follows. First, for young adults, as the probability of target presentation increases, the preceding time of the triceps surae activation with respect to the anterior deltoid (AD) activation would be longer and become closer to that in the self-timing task, with shorter latency and smaller amplitude of P300. Second, for older adults, ES activation would be earlier than AD activation, but the triceps surae activation would not be earlier than AD activation even in the self-timing task. Onset time of postural muscle activation, and latency and amplitude of P300 would be unaffected by target probability in the oddball task, and onsets of the triceps surae would be markedly later than in the self-timing task.

## Methods

### Subjects

Subjects comprised 14 young adults (7 men, 7 women) and 14 older adults (11 men, 3 women). Mean values (standard deviation (SD)) for age, height, weight, and foot length were 22.4 (1.9) years, 163.7 (5.5) cm, 57.0 (8.1) kg, and 24.4 (1.2) cm for young adults, and 70.3 (5.7) years, 156.3 (9.0) cm, 56.2 (7.3) kg and 23.6 (1.4) cm for older adults. Subject health status was assessed from a questionnaire. All subjects reported no history of neurological or orthopedic impairment and had normal hearing. Older subjects were community-dwelling individuals who could walk independently and perform activities of daily living without assistance. In accordance with the Declaration of Helsinki, all subjects provided informed consent after receiving an explanation of the experimental protocol, which was approved by the ethics committee at Kanazawa University.

### Apparatus

**Fig. 1:**
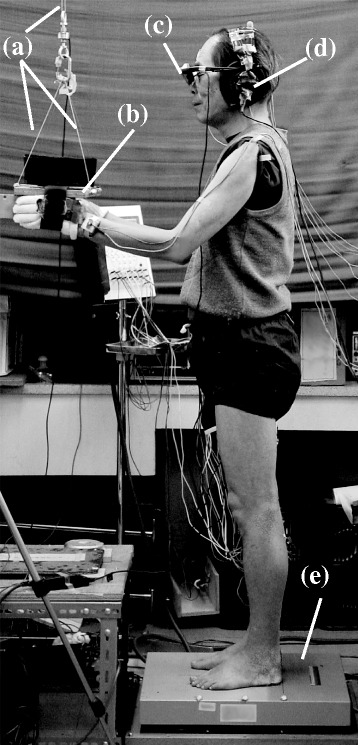
Experimental setup. **a** Metal wires. **b** Wooden board. **c** Eye-trek face-mounted display. **d** Headphones. **e** Force platform

To measure the center of pressure in the anteroposterior direction (CoPap), a force platform (OR6–6; AMTI, USA) was used (Fig. 1). CoPap electronic signals were sent simultaneously to three devices, a computer (PC9801BX; NEC, Japan) to determine CoPap position, another computer (Dimension E521; Dell Japan, Japan) for analysis, and an oscilloscope (DS6612; Iwatsu, Japan) to monitor the results. The onset time of postural muscle activation is influenced by CoPap position just before arm flexion [29]. To control initial CoPap positions, the first computer, which received CoPap data via an analog-to-digital (A/D) converter (PIO9045; I/O-Data, Japan) with a sampling rate of 20 Hz and 12-bit resolution, generated a buzzing sound when CoPap was located within a range of ± 1 cm of the quiet standing posture (QSP range). Since the SD for CoPap fluctuation during QSP for 60 s was approximately 0.5 cm for young healthy adults [30], the QSP range corresponds to ± 2 SDs of the fluctuation.

Subjects held wooden grips attached beneath a wooden board (17 cm × 37 cm × 1.7 cm; weight, 0.7 kg), with hands fixed to the board by acrylic belts (Fig. 1). The board was suspended by non-extensible metal wires (length, 70 cm) from a metal frame set outside the force platform. Total weight of the board was set at 3% of body weight using free weights. To ensure the subject maintained a standing posture without leaning on the board, force applied to the board by the subject was monitored using a load cell (LUB-B-50 KB; Kyowa, Japan) attached to the connection between the frame and wire. The validity of this apparatus for arm flexion with hands suspended has been verified [17]. In the arm flexion task, EEG activity is reported to be minimally contaminated by arm flexion electromyogram (EMG) activity [18].

A fixation point was presented on the center of an eye-trek face-mounted display (FMD011F; Olympus, Japan) (Fig. 1). In oddball tasks, auditory stimuli of 2000 and 1000 Hz were used as the target and non-target stimuli, respectively. Stimuli were delivered in random order via headphones with intensity of 90 dB, duration of 100 ms and inter-stimulus interval of 2.5 s (Synax; NEC, Japan). Probabilities of target presentation were set at 15 and 45%.

Surface electrodes (P-00-S; Ambu, Denmark) were used in bipolar derivation to record EMG activity of the following muscles: AD as a focal muscle for arm flexion; and ES at the level of the iliac crest, long head of biceps femoris (BF) at the midpoint between the ischial tuberosity and head of the fibula, and medial head of gastrocnemius (GcM) as postural muscles. Electrode location for both AD and GcM was the midportion of the muscle belly. Since the direction of arm movement (i.e., postural disturbance) was forward only and preceding activation of postural muscles was observed mainly in dorsal muscles [18], only dorsal postural muscles were selected. In the preliminary experiment, we also confirmed that activation of frontal postural muscles was small and did not occur earlier than activation of AD. Electrodes were placed on the right side of the body with an inter-electrode distance of about 3 cm. A ground electrode was placed over the right lateral malleolus. These electrodes were fixed after shaving and cleaning the skin with alcohol.

Ag-AgCl cup electrodes (diameter, 8 mm) for recording EEG were affixed to the scalp at Fz, Cz, and Pz in accordance with the international 10–20 system and referred to linked earlobes. A ground electrode was placed at Fpz. An electrooculogram (EOG) was recorded from a pair of electrodes placed immediately above and below the right eye.

Electrode input impedance was reduced to < 5 kΩ. Signals from electrodes were amplified (EMG × 4000, EEG × 10,000, EOG × 1000) and band-pass filtered (EMG 1.6–500 Hz, EEG 0.05–100 Hz, EOG 0.05–30 Hz) using an amplifier (EMG BIOTOP-6R12; NEC-Sanei, Japan; EEG and EOG: Synax; NEC, Japan). All electrical signals were sent to the computer for analysis via A/D converters (ADA16-32/2(CB) F; Contec, Japan) with a sampling rate of 1000 Hz and 16-bit resolution.

### Procedure

All measurements were performed on the force platform while standing barefoot with feet 10 cm apart and parallel, hands suspended 10 cm below the shoulder joints, and elbows flexed 60° (Fig. 1). Subjects were instructed to keep the shoulder muscles as relaxed as possible, not to lean toward the board and to gaze at the fixation point during all measurements. EMG activity in AD and force applied to the board were monitored by the experimenter and adjusted by oral instruction. Mean CoPap was initially measured 5 times for 10 s while the subject maintained QSP. The mean value from five measurements was adopted as the QSP position.

Next, bilateral arm flexion trials under the self-timing condition and the oddball task with 15 and 45% target probability were commenced. Subjects initially maintained CoPap position within the QSP range for at least 3 s while hearing the buzzing sound, which was then stopped by an experimenter. Under the self-timing condition, within 3 s of cessation of the buzzing sound, the subject initiated bilateral arm flexion at their own timing. Trials were repeated with a 30-s rest period between trials until 20 trials were accepted. In the oddball task, a 3-min experimental block comprising 72 target or non-target auditory stimuli started 3 s after cessation of the buzzing sound and the subject was required to respond only to the target stimuli. Experimental blocks were repeated with 3-min seated rest periods until 20 target trials per condition were accepted. Under all conditions, subjects were instructed to flex the arms at maximum speed, stop voluntarily at shoulder level, and maintain this position for 1 s before returning to the starting position. Trials in which the CoPap position just before arm flexion was beyond the QSP range were excluded from the count of acceptable trials. In the oddball task, trials with eye blinks and excessive muscle-related potentials (voltage on EOG or any EEG electrodes exceeding ± 100 μV) during the period from 200 ms before to 800 ms after target stimulus onset were also excluded. The order of conditions was randomized for each subject. Subjects were given 3 min of seated rest between conditions.

### Data analysis

All data were analyzed while blinded to subject condition, using BIMUTAS II signal analysis software (Kissei Comtec, Japan).

**Fig. 2:**
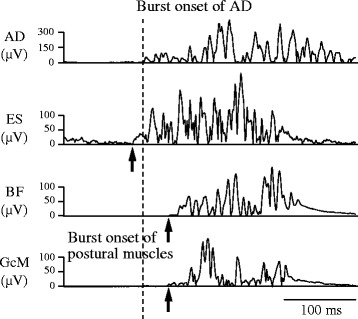
Representative waveforms of electromyogram data from an older subject performing the oddball task with 15% target probability. *AD* anterior deltoid, *ES* erector spinae, *BF* biceps femoris, *GcM* gastrocnemius

EMGs were analyzed as described below with reference to previous studies [16–18] (Fig. 2). To exclude electrocardiographic and movement artifacts, all EMGs were high-pass filtered at 40 Hz using a seventh-order Butterworth method, then full-wave rectified. Mean and SD of the amplitude for background activity of each muscle was calculated during the period from − 150 to 0 ms with respect to target onset for AD and from − 300 to − 150 ms with respect to burst onset of AD for postural muscles. Burst activation of each muscle was identified when onset was within + 200 to + 500 ms after target onset for AD and − 150 to + 100 ms with respect to burst onset of AD for postural muscles, and when the envelope line of the burst activity deviated more than the mean + 2 SDs from background activity for at least 50 ms. Burst onset was defined as the time point at which the above deviation began in the EMG wave included in the envelope line. The onset time of postural muscles was defined as the time difference between burst onsets of postural muscles and AD and presented as a negative value when burst onset of postural muscles preceded AD. Mean values for the onset time of each postural muscle were calculated separately for all conditions and used as representative values for subjects.

**Fig. 3:**
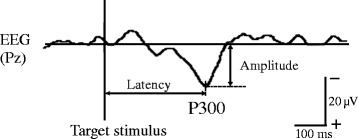
Representative waveform of P300 component of a young subject performing the oddball task with 15% target probability. *EEG* electroencephalogram

The P300 component is elicited by target stimuli in a discrimination task [25] and show maximum amplitude in the parietal area [31]. In the oddball task, therefore, waveforms for target stimuli recorded from Pz were averaged separately for each condition and analyzed for P300 (Fig. 3). Twenty trials were adopted for each averaging. Averaged epochs extended from 200 ms before to 800 ms after target stimulus onset. Mean amplitude during the 200-ms pre-stimulus period was defined as the baseline for averaging. The averaged waveform was smoothed using a 30-Hz low-pass filter. The largest positive peak between 250 and 500 ms after target stimulus onset was defined as P300 with reference to previous studies [17, 21, 26, 28]. Latency and amplitude of the peak were calculated as the time from onset of target stimulus to the peak and the voltage difference from baseline to peak, respectively.

### Statistical analysis

The Shapiro-Wilk tests confirmed that all data satisfied the assumptions of a normal distribution. A one-sample *t* test was used to assess whether burst onset of the postural muscles differed significantly from that of AD. The following analyses were separately used for subject groups. After Levene’s tests confirmed that the variances of onset times of each postural muscle were equal in every condition, two-way mixed factorial analysis of variance was used to assess the effects of condition (15%, 45%, and self-timing) and muscle (ES, BF, and GcM) on the onset time of postural muscle activation, with repeated measures on the condition factor. Greenhouse-Geisser correction was applied when Mauchly’s test of sphericity was not met. When a significant interaction between these effects or a main effect of condition or muscle was shown, post hoc multiple-comparison analyses using Tukey’s honestly significant difference were performed separately to assess differences among conditions and muscles. A paired *t* test was used to assess differences in latency and amplitude of P300 between conditions in oddball tasks (15 and 45%). Pearson correlations were used to evaluate the magnitude of correlation in oddball tasks between latency or amplitude of P300 and onset time of each postural muscle activation, and between latency and amplitude of P300. For each onset time of postural muscles under each condition, Student’s *t* test was used to investigate differences between young and older adults, after an *F* test was used to confirm whether variance was equal. Welch’s correction was applied when equal variance was rejected. Alpha level was set at *p* < 0.05. All statistical analyses were performed using IBM SPSS Statistics version 19 (IBM Japan, Japan).

## Results

**Fig. 4:**
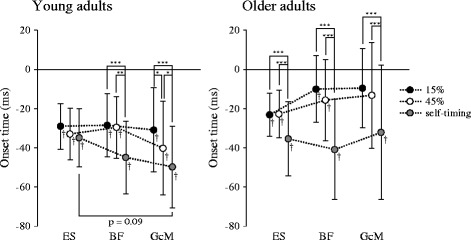
Means and standard deviations for onset time of postural muscle activation relative to onset of anterior deltoid (AD). *ES* erector spinae, *BF* biceps femoris, *GcM* gastrocnemius. *p < 0.05, **p < 0.01, ***p < 0.001. † Significant differences between burst onsets of AD and each postural muscle (*p* < 0.05)

Figure 4 shows mean and SD for onset time of postural muscles under each condition. In young adults, all postural muscles were activated significantly earlier than AD under each condition (*t*_13_ > 5.4, *p* < 0.001). A significant interaction between condition and muscle was found for the onset time of postural muscles in young adults (*F*_3.2,61.5_ = 3.1, *p* < 0.05). For the significant differences in onset time among conditions, ES showed no differences, BF was earlier in the self-timing condition than in 15 and 45% conditions (*p* < 0.01), and GcM was earlier in the order of self-timing, 45 and 15% conditions (*p* < 0.05). In the self-timing condition, onset time tended to be earlier in GcM than in ES (*p* = 0.09). In 15 and 45% conditions, no significant differences were found in onset time among muscles. In older adults, activation significantly preceding AD was observed in all muscles under the self-timing condition (*t*_13_ > 3.5, *p* < 0.01), and in ES and BF under the 15 and 45% conditions (*t*_13_ > 2.2, *p* < 0.05). However, preceding activation was not found in GcM under the 15 and 45% conditions. Only a significant main effect of condition was found for onset time of postural muscle activation in older adults (*F*_1.5,58.4_ = 28.2, *p* < 0.001). Onset time of all muscle activations was significantly earlier under the self-timing condition than under 15 and 45% conditions (*p* < 0.001), and no significant difference was found between 15 and 45% conditions. Only for the onset time of GcM in the self-timing task, variance was significantly larger in older adults than in young adults (*F*_13,13_ = 2.7, *p* < 0.05). Onset times of BF and GcM in the 15% condition and ES and GcM in the 45% condition were significantly later in older adults than in young adults (*t*_26_ > 2.1, *p* < 0.05). No significant differences were found between young and older adults in onset time of ES in 15% condition, BF in 45% condition, and all postural muscles in the self-timing condition.

**Fig. 5:**
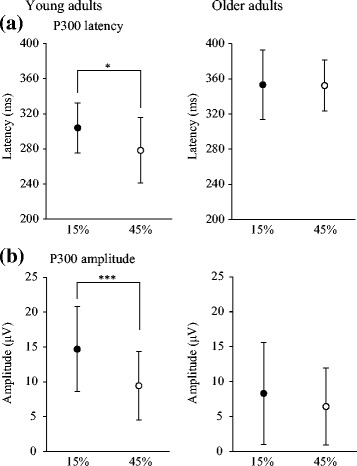
Means and standard deviations for latency (**a**) and amplitude (**b**) of P300. 15%: oddball task with 15% target probability; 45%: oddball task with 45% target probability. *p < 0.05, ***p < 0.001

Figure 5 shows mean and SD for latency and amplitude of P300 in 15 and 45% conditions. In young adults, latency and amplitude of P300 were significantly shorter and smaller under the 45% condition than under the 15% condition (latency *t*_13_ = 2.7, amplitude; *t*_13_ = 5.6, *p* < 0.05). In older adults, no significant differences in latency or amplitude of P300 were found between conditions.

In both young and older adults, no significant correlations were found between latency or amplitude of P300 and onset time of each postural muscle activation, or between latency and amplitude of P300.

## Discussion

In the self-timing task for young adults, all postural muscles activated significantly earlier than AD, and the preceding time tended to be longer in GcM than in ES (Fig. 4). Older adults also showed preceding activation of all postural muscles, but no significant differences were seen among muscles in terms of preceding time (Fig. 4). Our result revealed that older adults could use the ankle joints in addition to the trunk and hip joints for postural control during arm flexion with sufficient postural preparation. However, the focus was not wholly on the ankles. No significant differences were found between young and older adults in onset times of all postural muscles in this task. However, only for GcM, variance of onset time was significantly larger in older adults than in young adults. These results probably indicated that some older adults would show later onset time of GcM. This suggests that age-related deterioration, in the form of reduced use of the ankle joints, would occur in behavior processing for postural control also in the arm movement task, as reported in other postural control tasks [1, 4–6].

In the oddball task for young adults, all postural muscles activated earlier than AD in both 15 and 45% conditions, and no significant differences in preceding time were seen among muscles (Fig. 4). Onset time of GcM activation in the 45% condition was significantly earlier than in the 15% condition but later than in the self-timing task. In this task, cognitive processing of stimuli is necessary in addition to behavior processing and these processes are presumably executed in parallel [32]. These findings suggest that when target probability increases, young adults could predict the presentation timing of target stimuli to some extent, resulting in a longer preceding time of GcM activation. With a higher probability of target presentation, P300 latency became shorter and P300 amplitude became smaller (Fig. 5), consistent with previous findings [22, 23, 26, 27]. Latency and amplitude of P300 indicate the cognitive processing time and allocation of attention to the processing, respectively [21–23]. This indicates that when the target presentation timing could be predicted with higher probability, young adults may perform cognitive processing more rapidly despite less attentional allocation to the processing. This would result in more sufficient postural preparation and earlier onset of activation of the postural muscles in the lower legs. However, changes in onset time of postural muscle activation, especially for GcM, did not show significant correlations with changes in latency or amplitude of P300. Furthermore, no significant correlation was found between changes in latency and amplitude of P300. Predictability may strongly affect cognitive processing time for some subjects and the amount of attentional allocation to this processing for other subjects. Such individual differences would result in an inconsistent relationship between changes in cognitive processing and onset time of postural muscle activation with higher predictability.

For older adults, preceding activation was found in ES and BF, but not in GcM in the oddball task, regardless of the probability. No significant differences in onset time of all postural muscles were seen between 15 and 45% conditions (Fig. 4). This postural muscle pattern is similar to the pattern when young adults perform arm flexion from a suspended posture in response to a stimulus that appears suddenly [16]. The comparison between postural control patterns in the self-timing and oddball tasks suggests that older adults could not perform postural control primarily using the ankle joints because of deterioration of cognitive function. The latency and amplitude of P300 for older adults also showed no significant differences between 15 and 45% conditions (Fig. 5). When target stimuli were presented in fixed or random order with a probability of 20%, young adults showed significantly larger P300 amplitude elicited by target stimuli in random order than that in fixed order, but older adults showed no significant differences between P300 amplitudes elicited by targets in fixed and random order [33]. Moreover, compared to young adults, older subjects have shown significantly reduced cortical activation in bilateral superior temporal gyri in an oddball task, which has been interpreted as representing declines in auditory sensory memory and automatic change detection [34]. These findings suggest that older adults would have difficulty predicting the presentation timing of target stimuli based on the sequence of target and non-target stimuli. Furthermore, attentional allocation is reported to be changed according to the significance of the processing to carry out each task [35]. Compared with young adults, postural control for older adults is reported to be strongly impaired by an additional cognitive task during postural control task [36, 37]. Older adults in the present study might also allocate substantial attention not to behavior processing related to postural control but rather to cognitive processing of stimuli because of difficulty predicting the presentation timing of target stimuli. Therefore, in the oddball task, such difficulty would lead to insufficient postural preparation and reduced use of the ankle joints.

The present study could not demonstrate which age-related deficits of multiple cognitive aspects, including memory, attention, information processing, and executive function [38], would strongly affect postural controllability, or how these deficits might be related to each other and postural controllability. In future studies, we will set cognitive tasks to separately control for each cognitive aspect and will investigate the relationship between age-related changes in each cognitive function and postural controllability in older adults.

## Conclusions

During arm movement, young adults can achieve sufficient postural preparation in proportion to the probability of target presentation in the oddball task. Older adults can achieve postural control using ankle joints in the self-timing task. However, in the oddball task, older adults experience difficulty predicting the timing of target presentation, which could be related to deteriorated cognitive function, resulting in reduced use of the ankle joints for postural control.

## Abbreviations

A/D: Analog-to-digital
AD: Anterior deltoid
BF: Biceps femoris
CoPap: Center of pressure in the anteroposterior direction
EEG: Electroencephalogram
EMG: Electromyogram
EOG: Electrooculogram
ES: Erector spinae
GcM: Gastrocnemius
QSP: Quiet standing posture
SD: Standard deviation
